# Clinical 3-D Gait Assessment of Patients With Polyneuropathy Associated With Hereditary Transthyretin Amyloidosis

**DOI:** 10.3389/fneur.2020.605282

**Published:** 2020-11-23

**Authors:** Maria do Carmo Vilas-Boas, Ana Patrícia Rocha, Márcio Neves Cardoso, José Maria Fernandes, Teresa Coelho, João Paulo Silva Cunha

**Affiliations:** ^1^INESC TEC, FEUP and LABIOMEP, University of Porto, Porto, Portugal; ^2^Unidade Corino de Andrade and Neurophysiology Department, Centro Hospitalar Universitário do Porto, Porto, Portugal; ^3^Institute of Electronics and Informatics Engineering of Aveiro (IEETA), Department of Electronics, Telecommunications and Informatics, University of Aveiro, Aveiro, Portugal

**Keywords:** ATTRv V30M, amyloidosis, polyneuropathy, gait, quantitative assessment, ambulatory, markerless vision-based systems

## Abstract

Hereditary amyloidosis associated with transthyretin V30M (ATTRv V30M) is a rare and inherited multisystemic disease, with a variable presentation and a challenging diagnosis, follow-up and treatment. This condition entails a definitive and progressive motor impairment that compromises walking ability from near onset. The detection of the latter is key for the disease's diagnosis. The aim of this work is to perform quantitative 3-D gait analysis in ATTRv V30M patients, at different disease stages, and explore the potential of the obtained gait information for supporting early diagnosis and/or stage distinction during follow-up. Sixty-six subjects (25 healthy controls, 14 asymptomatic ATTRv V30M carriers, and 27 symptomatic patients) were included in this case-control study. All subjects were asked to walk back and forth for 2 min, in front of a Kinect v2 camera prepared for body motion tracking. We then used our own software to extract gait-related parameters from the camera's 3-D body data. For each parameter, the main subject groups and symptomatic patient subgroups were statistically compared. Most of the explored gait parameters can potentially be used to distinguish between the considered group pairs. Despite of statistically significant differences being found, most of them were undetected to the naked eye. Our Kinect camera-based system is easy to use in clinical settings and provides quantitative gait information that can be useful for supporting clinical assessment during ATTRv V30M onset detection and follow-up, as well as developing more objective and fine-grained rating scales to further support the clinical decisions.

## Introduction

Hereditary amyloidosis associated with transthyretin (ATTRv amyloidosis) is a highly disabling multisystemic disorder with autosomal dominant inheritance and variable penetrance (1). The most frequent mutation, causing important foci of the disease in several countries or regions (e.g., Portugal, Sweden, Japan, Brazil), shows the replacement of valine by methionine at position 30 of the TTR protein (TTRVal30Met). Portugal harbors the largest known clusters of patients, with recently reported prevalence of 22.93/100,000 adult inhabitants (2).

The disease described in Portugal presents as a length-dependent symmetric polyneuropathy that typically starts in the feet with loss of temperature and pain sensations. It is associated to life-threatening autonomic dysfunction, leading to cachexia and death within 7.3 to 11 years from onset, if left untreated (3). Onset occurs typically around 35 years old (4), although in some areas, namely Sweden and Japan, patients present a late onset—age of onset around 50 years old or older—which presents a faster and more severe disease progression (5) and less autonomic involvement compared to early onset cases.

As in diabetic neuropathy [one of the most common neuropathies in the western world (6)], the first clinical characteristics of the disease's evolution are usually positive (burning sensations, pain, pricking, tingling) and negative (anesthesia and analgesia, hypoesthesia and hypoalgesia) sensory symptoms in the limbs (7). The natural course of this condition is classically classified into three stages: I—patients are ambulatory, have mostly mild sensory, motor, and autonomic neuropathy in the lower limbs; II—they are still ambulatory but require assistance and have mostly moderate impairment progression to the lower limbs, upper limbs, and trunk; and III—bedridden or wheelchair bound and present severe sensory, motor, and autonomic involvement of all limbs (4).

Even though ATTRv V30M was first described in 1952 (8), current management strategies lack cohesion and patients experience years of misdiagnosis and negligible treatment (3). This polyneuropathy is currently evaluated clinically, with a complete anamnesis and neurologic examination (including visual evaluation of gait), and neurophysiologically, with nerve conduction studies (NCS), sympathetic skin response (SSR), and quantitative sensory testing (QST) (9). New therapeutic strategies are under development (10) and gene modifying drugs have been released to the market, such as antisense oligonucleotides (inotersen) (11) and small interfering RNAs (patisiran) (12).

Nowadays, available vision-based systems allow capturing human motion in 3-D, providing quantitative information regarding motion, which can be valuable for supporting the assessment of patients with movement/gait impairments. The Kinect camera is a “red-green-blue-depth” (RGB-D) camera, which is able to detect people's silhouettes and estimate the 3-D position of a person's body joints, relying on the depth map of the space in front of it using information obtained by its infrared sensor (13). This type of camera has shown to have the potential to be an adequate solution for supporting patient physical function evaluation in clinical settings or at home (14–16).

In contrast with reference systems traditionally used for quantitative motion analysis (multi-camera marker-based systems deployed in an especially dedicated laboratory), RGB-D camera-based systems are low-cost, easy to set up and minimally intrusive. Despite its lower precision comparing to the reference systems, the validity of the Kinect for assessing clinically relevant movements, including gait, has already been studied either with healthy or impaired populations (17, 18). Galna et al. showed that the Kinect is able to accurately measure timing and spatial characteristics and therefore provide valuable knowledge in the context of motor disorders evaluation (18).

In this contribution, which builds on our previous work where we studied the validity of our gait analysis system based on a RGB-D camera in the context of ATTRv V30M patient assessment and disease progression evaluation (15, 16), we aim to characterize the influence that ATTRv V30M polyneuropathy may have in patients' gait and, if any differences are detected, verify if the changes have any diagnostic or group-distinction value. We used the vision-based system developed by our group, which includes a Kinect camera, to gather 3-D body data from healthy controls, asymptomatic carriers, and ATTRv V30M patients with different degrees of disease evolution, while they performed a gait task. We then performed automated gait analysis, where several gait parameters were computed. To verify if the obtained quantitative information is valuable for supporting ATTRv V30M clinical gait assessment, we investigated if there were differential gait characteristics which may aid the diagnosis of the polyneuropathy, prediction of motor impairment onset and/or distinction among different disease phases.

## Materials and Methods

### Subjects

This experiment was carried out at Hospital de Santo António, Centro Hospitalar Universitário do Porto, Portugal, with the participation of 66 subjects:

- 25 healthy controls (HC);
- 14 asymptomatic carriers of the V30M mutation (AC);
- 27 symptomatic patients (SP).

This study was authorized by the local Ethics Committee, complies with the Declaration of Helsinki, and all subjects signed an informed consent form. The exclusion criteria for this study were the presence of orthopedic comorbidities of the lower limbs, and other neurological conditions.

Healthy controls were chosen from the university and hospital staff and presented no complaints, symptoms, or history related to polyneuropathy. All patients were selected by a neurologist, and did not have a clear risk factor for diabetic neuropathy, alcoholism, cancer and autoimmune diseases.

Since the symptomatic patients' group was composed by patients with very heterogeneous clinical status, after their first analysis, these patients were divided in the following three subgroups:

- SPS: patients with small-fiber neuropathy signs and symptoms on neurological examination or neurophysiological tests, without any large-fiber involvement on neurological examination (no vibratory or proprioceptive abnormalities, no motor weakness) or on neurophysiological tests (normal NCS, normal QST for vibration).
- SPSL: patients with small-fiber neuropathy signs and symptoms, and some large-fiber sensory abnormality on neurological examination or neurophysiological tests (QSTs or sensory NCS), with no muscular weakness on neurological examination or abnormalities on motor nerve conduction studies.
- SMP: patients with small-fiber neuropathy signs and symptoms, with sensory large fiber involvement, and also with some distal muscular weakness or motor NCS abnormalities.

The asymptomatic carriers showed normal neurological examination and no alteration of the QST, NCS, heart rate deep breathing and SSR.

The demographic data (gender, and mean ± standard deviation as well as minimum and maximum values, for age, height and weight) and neuropathy impairment score (NIS) of all participants are presented in Table 1.

**Table 1:** Subjects' characterization by assessed group: gender, average ± standard deviation (minimum, maximum) for age, height and weight, and neuropathy impairment score (NIS).

**Subject group**	**Gender**	**Age, years**	**Height, cm**	**Weight, kg**	**NIS**
1. HC (25)	13 M/12 F	30.3 ± 7.8	171.6 ± 10.6	69.8 ± 15.8	0
		(19, 51)	(150, 194)	(48, 105)	
2. AC (14)	8 M/6 F	33.6 ± 7.9	167.8 ± 8.8	68.5 ± 8.9	0
		(23, 54)	(154, 184)	(51, 80)	
3. SP (27)	13 M/14 F	40.85 ± 10.48	169.3 ± 9.7	66.0 ± 12.1	12.1 ± 20.9
		(23, 63)	(149, 186)	(46, 101)	(0, 91)
3.1 SPS	6 M/9 F	36.6 ± 7.9	169.7 ± 9.6	66.7 ± 11.4	3.6 ± 3.6
		(25, 56)	(156, 185)	(51, 101)	(0, 12)
3.2 SPSL (7)	3 M/4 F	42.0 ± 10.5	166.7 ± 7.2	61.6 ± 12.9	10.3 ± 9.7
		(23, 55)	(155, 180)	(46, 80)	(0, 28)
3.3 SMP (5)	4 M/1 F	52.0 ± 8.4	171.4 ± 12.3	70.0 ± 11.3	46.9 ± 33.9
		(37, 63)	(149, 186)	(55.8, 82)	(12, 91)

### Experimental Setup and Protocol

**Figure 1:**
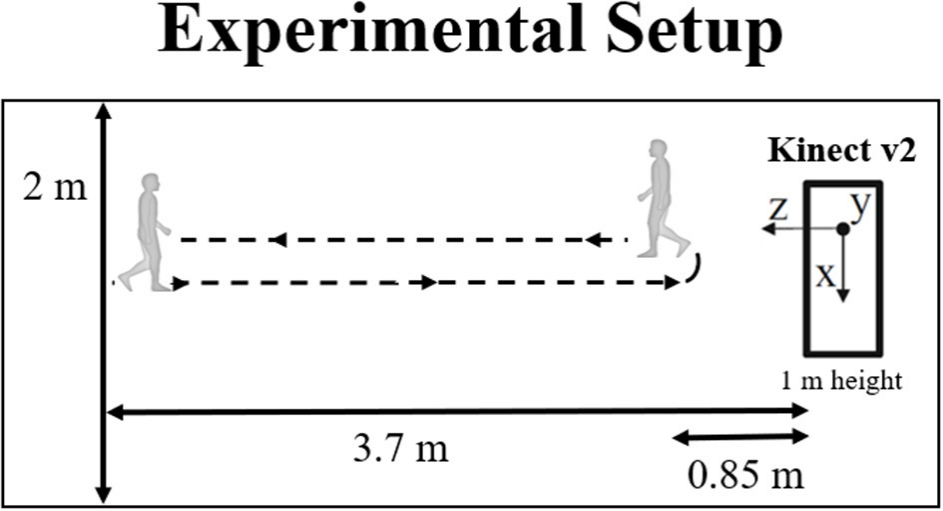
Experimental setup used for data acquisition, including the relevant distances and the coordinate system associated with the Kinect v2.

The experiment took place at the hospital's Neurophysiology Department. The setup included our gait analysis system, including an RGB-D camera (in this case, the Kinect v2), which was used to acquire depth, infrared and 3-D body joint data from the subjects. Their task was simply walking during 2 min at their natural pace (using their usual walking shoes), according to the trajectory represented in Figure 1.

The Kinect was placed at a height of approximately 1 m, and its tilt angle was varied, according to the subject's height, to maximize the practical depth range (i.e., the range for which the camera is able to track all body joints) for each person. All the relevant distances, as well as the 3-D coordinate system associated with the Kinect, are also represented in Figure 1.

### Data Processing

The data were acquired at 30 Hz and processed as described in (19). Each body data frame includes the 3-D position of the joints tracked by the Kinect [see (19) for the joint list].

**Figure 2:**
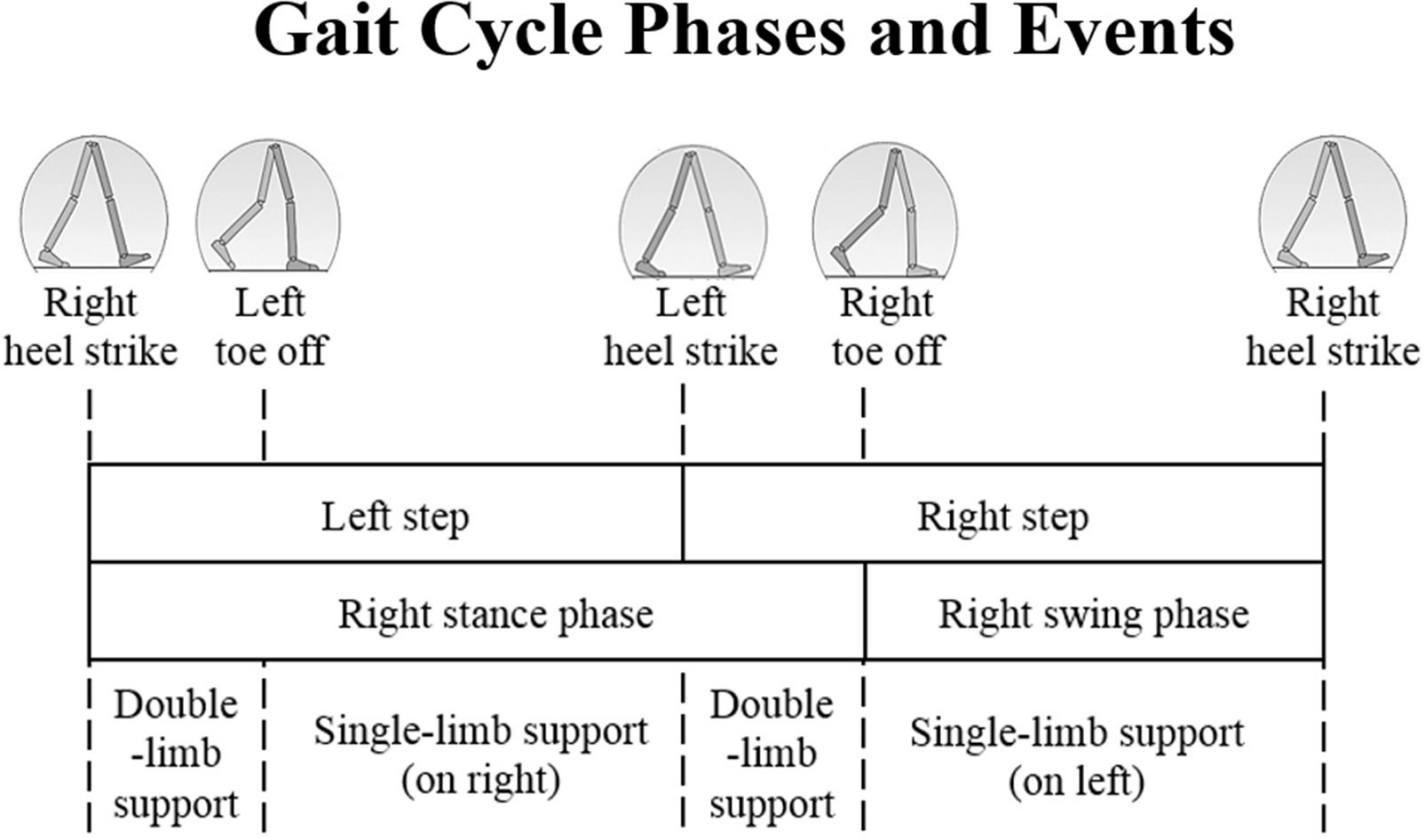
Gait cycle phases and positions of the legs during a single gait cycle associated to the right leg (darker), adapted from (20).

The time intervals corresponding to walking toward the camera were automatically selected according to (19) and then matched with the different gait cycle phases shown in Figure 2. One gait cycle includes three consecutive heel strikes, starting and ending with a heel strike associated with the same foot. It also includes two toe-off events, which occur between two consecutive heel strikes of different feet. The detection of heel strikes and toe offs were performed as described in (19) and (15), respectively.

For each gait cycle, we computed the 24 spatiotemporal and kinematic gait parameters listed in Table 2 and defined in (15). The total body center of mass (TBCM) sway was computed as the standard deviation of the distance (in the x/y-axis, i.e., medial-lateral and vertical directions) of the total body center of mass (TBCM), in relation to the RGB-Dsensor's coordinate system, for all gait cycle frames. For each frame, TBCM's position is the mean position of all body segments' CM, which was obtained according to (21).

**Table 2:** Mean ± standard deviation values for each gait parameter and each subject group (1. Healthy Controls – HC; 2. Asymptomatic Carriers – AC; 3. Symptomatic Patients – SP; 3.1 Patients with small-fiber sensory polyneuropathy – SPS – and 3.2 with large-fiber sensory polyneuropathy – SPSL; and 3.3 Patients with motor neuropathy – SMP).

**Gait parameter**	**1. HC**	**2. AC**	**3. SP**	**3.1 SPS**	**3.2 SPSL**	**3.3 SMP**
Stride duration, s	1.238 ± 0.386	1.463 ± 0.518	1.628 ± 0.690	1.709 ± 0.729	1.463 ± 0.509	1.616 ± 0.768
Stride length, cm	114.7 ± 23.0	101.5 ± 28.0	93.6 ± 25.5	92.8 ± 25.6	91.7 ± 23.7	100.2 ± 27.3
Step duration, s	0.626 ± 0.282	0.732 ± 0.343	0.823 ± 0.489	0.862 ± 0.517	0.744 ± 0.396	0.816 ± 0.513
Step length, cm	52.2 ± 13.5	47.3 ± 15.5	42.6 ± 15.6	42.6 ± 15.9	41.6 ± 15.3	44.7 ± 14.4
Step width, cm	12.4 ± 3.7	12.1 ± 3.8	13.0 ± 4.2	13.7 ± 4.4	12.1 ± 4.1	12.2 ± 3.2
Stance duration, s	0.784 ± 0.261	0.943 ± 0.396	1.012 ± 0.525	1.057 ± 0.559	0.901 ± 0.360	1.044 ± 0.608
Swing duration, s	0.455 ± 0.245	0.521 ± 0.301	0.616 ± 0.414	0.652 ± 0.451	0.562 ± 0.342	0.573 ± 0.367
Single support duration, s	0.897 ± 0.331	1.043 ± 0.451	1.219 ± 0.575	1.292 ± 0.623	1.104 ± 0.440	1.142 ± 0.559
Double support duration, s	0.341 ± 0.125	0.421 ± 0.172	0.409 ± 0.267	0.417 ± 0.296	0.359 ± 0.139	0.474 ± 0.309
Gait speed, m/s	1.047 ± 0.239	0.846 ± 0.228	0.728 ± 0.180	0.696 ± 0.161	0.767 ± 0.182	0.785 ± 0.218
Gait speed variability, m/s	0.105 ± 0.057	0.153 ± 0.384	0.121 ± 0.216	0.107 ± 0.119	0.125 ± 0.208	0.170 ± 0.423
Foot swing velocity, m/s	2.679 ± 1.031	2.318 ± 1.334	1.894 ± 1.109	1.807 ± 1.103	1.917 ± 0.917	2.195 ± 1.376
Arm swing velocity, m/s	1.976 ± 0.749	1.570 ± 0.798	1.384 ± 0.709	1.312 ± 0.515	1.405 ± 0.575	1.633 ± 1.299
Total body center of mass sway in *x-axis*, cm	29.2 ± 38.7	35.5 ± 26.1	34.1 ± 37.9	29.7 ± 33.1	44.7 ± 41.5	31.5 ± 44.2
Total body center of mass sway in *y-axis*, cm	10.2 ± 5.4	13.7 ± 15.5	11.1 ± 8.4	10.9 ± 7.5	11.6 ± 10.7	10.9 ± 7.0
Neck angle, deg	166.1 ± 10.2	162.3 ± 14.2	158.1 ± 17.7	160.4 ± 13.8	152.1 ± 23.5	160.2 ± 16.5
Spine shoulder angle, deg	171.3 ± 5.8	168.5 ± 8.7	165.7 ± 11.3	167.1 ± 8.9	161.9 ± 15.3	167.2 ± 9.4
Spine middle angle, deg	175.9 ± 2.2	174.7 ± 2.7	173.6 ± 3.3	173.8 ± 3.2	172.8 ± 3.6	173.9 ± 3.3
Maximum elbow angle, deg	167.6 ± 10.9	166.0 ± 9.7	166.0 ± 10.1	166.1 ± 9.3	166.5 ± 12.8	164.5 ± 6.2
Minimum elbow angle, deg	144.3 ± 20.4	144.7 ± 20.4	144.8 ± 21.8	145.3 ± 20.1	144.7 ± 26.0	142.6 ± 19.9
Maximum knee angle, deg	174.5 ± 3.1	176.8 ± 2.5	174.8 ± 3.5	174.5 ± 3.6	174.8 ± 3.4	176.1 ± 3.1
Minimum knee angle, deg	142.0 ± 17.7	142.5 ± 17.1	143.1 ± 18.4	142.6 ± 18.1	145.3 ± 17.7	141.1 ± 20.8
Hip angle range, deg	19.3 ± 7.0	18.9 ± 7.5	17.1 ± 6.8	17.3 ± 7.2	16.0 ± 5.8	18.4 ± 7.1
Ankle angle range, deg	33.6 ± 16.6	27.9 ± 17.6	20.7 ± 16.0	17.6 ± 14.2	22.0 ± 16.0	30.3 ± 18.4

### Statistical Analysis

To evaluate if the extracted gait parameters can be used to distinguish between the HC, AC and SP groups and also between the patient's subgroups (SPS, SPSL, and SMP), we performed the Kruskal–Wallis test (22) for each parameter. If the test's *p*-value was lower than or equal to the defined significance level of 0.05, we further performed the Conover-Iman test for multiple pairwise comparisons (23). All statistical analyses were performed in the R environment (version 3.5.1).

## Results

Table 2 presents the gait parameters' mean and standard deviation values for all the evaluated groups of participants: healthy controls (HC); asymptomatic carriers (AC); symptomatic patients (SP); patients with the clear involvement of small fibers (SPS) and with objective (clinical and neurophysiological) involvement of large fibers (SPSL); and patients with motor neuropathy (SMP).

**Figure 3:**
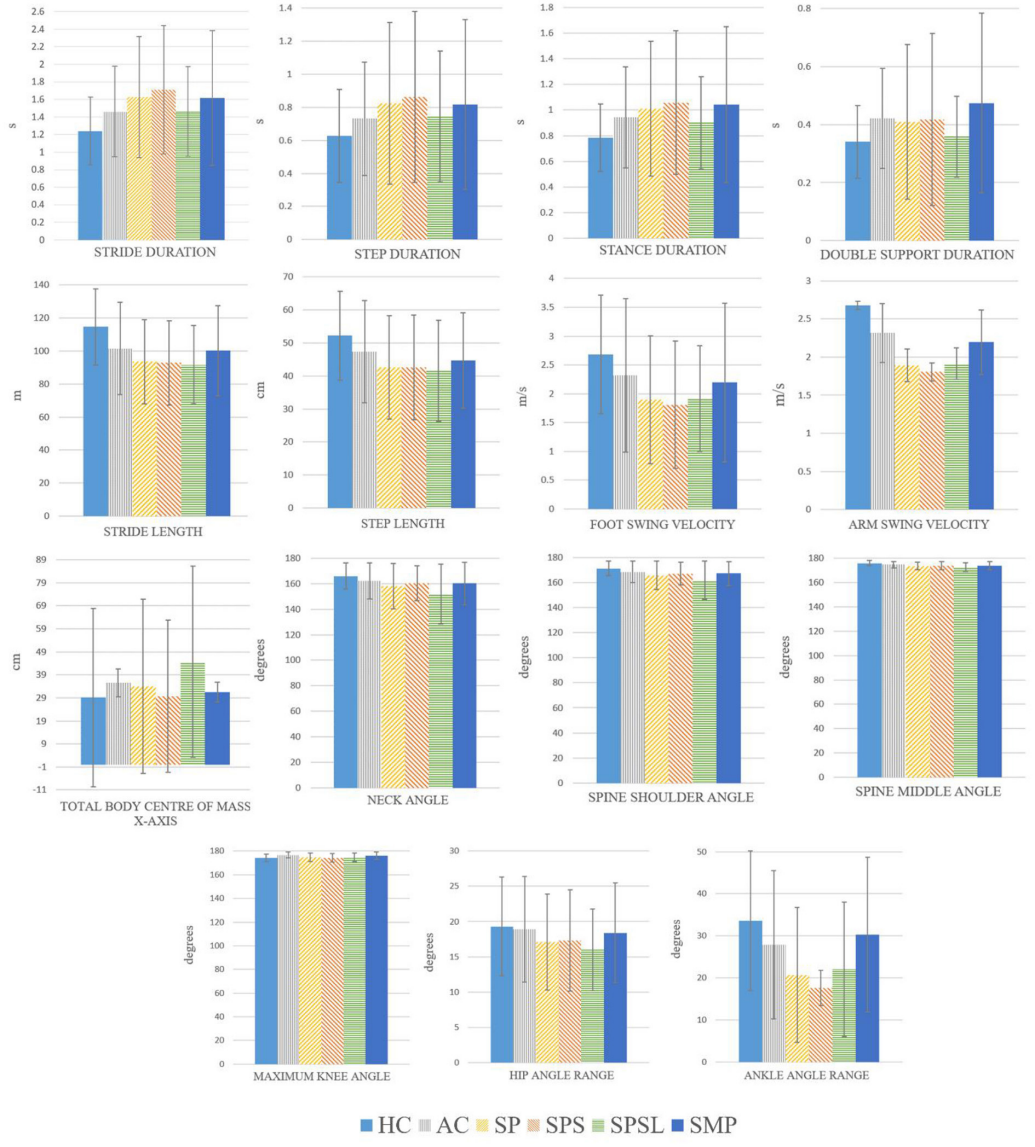
Mean and standard deviation for each subject group, for the gait parameters that showed statistically significant differences in the Conover-Iman test (p ≤ 0.05) for all the comparisons, when comparing different pairwise comparison, included in Table III, between the six groups (HC, AC, SP, SPS, SPSL and SMP).

The Kruskal–Wallis test results showed statistically significant differences (*p* ≤ 0.05) between the HC, AC and SP, as well as HC, AC, and SPS, SPSL, and SMP groups for all gait parameters. Therefore, we then performed the Conover–Iman test for the pairwise comparison between the analyzed groups, for each parameter. The analyzed group pairs that we considered most relevant, due to their clinical profile differences and amount of neurological deficits, are presented in Table 3. The remaining comparisons are presented in the Supplementary Material. Figure 3 shows the mean and standard deviation values for the parameters that showed statistically significant difference (*p* ≤ 0.05, CI 95%) for all pairwise comparisons indicated in Table 3.

**Table 3:** Results of the Conover-Iman test (*p*-value) pairwise comparison between the six groups included in Table 2 (HC, AC, SP, SPS, SPSL and SMP), for each gait parameter.

**Gait parameter**	**HC–AC**	**HC–SP**	**AC–SP**	**AC–SPS**	**SPS–SPSL**	**SPSL–SMP**	**HC–SMP**
Stride duration, s	≤ 0.001	≤ 0.001	≤ 0.001	≤ 0.001	≤ 0.001	≤ 0.001	≤ 0.001
Stride length, cm					0.04		
Step duration, s					≤ 0.001		
Step length, cm							
Step width, cm	N.S.					N.S.	N.S.
Stance duration, s	≤ 0.001					≤ 0.001	≤ 0.001
Swing duration, s						N.S.	
Single support duration, s							
Double support duration, s						≤ 0.001	
Gait speed, m/s						N.S.	
Gait speed variability, m/s		N.S.				≤ 0.001	
Foot swing velocity, m/s		≤ 0.001				0.002	
Arm swing velocity, m/s						≤ 0.001	
Total body center of mass sway in *x-axis* (TBCMx), cm							0.036
Total body center of mass sway in *y-axis* (TBCMy), cm	N.S.				N.S.		N.S.
Neck angle, deg	≤ 0.001				≤ 0.001		≤ 0.001
Spine shoulder angle, deg							
Spine middle angle, deg							
Maximum elbow angle, deg			N.S.	N.S.			
Minimum elbow angle, deg	N.S.		0.009	0.038	0.003		N.S.
Maximum knee angle, deg	≤ 0.001		≤ 0.001	≤ 0.001	0.046		≤ 0.001
Minimum knee angle, deg	0.007		N.S.	N.S.	0.002	0.022	N.S.
Hip angle range, deg	≤ 0.001		≤ 0.001	≤ 0.001	≤ 0.001	≤ 0.001	≤ 0.001
Ankle angle range, deg							

*N.S. stands for non-significant (p-value > 0.05)*.

## Discussion

Although we would expect asymptomatic patients to present similar characteristics in terms of gait when compared with healthy subjects—because the disease is not yet established—our study shows a statistically significant difference (*p* ≤ 0.05, CI 95%) among these two groups for several gait parameters (see Table 3). Despite being statistically significant, all these differences were undetected to the naked eye. If the obtained results correspond to a relaxed and natural data collection moment, the differences detected between these groups suggest an important subclinical disease onset.

On the other hand, some reasons may be drawn to explain the HC and AC group comparison's results. The AC subjects belong to a special group who knows they have the V30M mutation. Many of them have seen their family members deteriorating due to this disease, and may have been psychologically affected or stressed by the assessment, since it was performed at the hospital on the same day they were evaluated to check if they already manifested the disease. This may have affected their performance, since it is known that human gait is not an automatic task and the fact that emotions affect gait performance has been extensively described in literature (24–26). Studies have demonstrated that patients who perceive themselves as more disabled achieve lower levels of function (24). These descriptions are compatible with the differences noted between HC and AC in this study. Moreover, studies have shown that being a carrier of the V30M mutation, whether symptomatic or asymptomatic, is associated with more vulnerability to psychopathological symptoms and emotional distress (27), especially with the proximity to the age of onset which can trigger anxiety (28). In future gait analysis studies, the use of an anxiety measurement tool would therefore be very useful to complement the evaluation.

Patients in a more advanced stage of the disease, namely suffering of motor neuropathy (SMP) are possibly the ones suffering less influence of psychological factors since they are used to their condition. Also, these patients seem to compensate the steppage with a higher flexion of the hip and knee, and the foot drop is well-noticed by the ankle angle values in Table 2. It seems that patients find stabilization strategies (e.g., shorter swing phase) to deal with their difficulties, leading to having some gait characteristics closer to the asymptomatic carriers or healthy subjects than to patients without motor neuropathy (this happens for 7 out of the 24 parameters, 29%). It is important to note, however, that SPS, SPSL, and SMP groups have a lower sample size in our study.

When we look at the differences between asymptomatic carriers (AC) and symptomatic patients (SP), only the maximum elbow and minimum knee angles have a non-significant difference, which suggests that these parameters may not be related with disease onset. All other analyzed parameters show statistically significant differences between these two groups, namely in the direction of smaller and slower steps, less TBCM sway, and smaller angular movements for the SP group. These results may be explained by a more conscious gait, compatible with a greater attention to every movement, and are consistent with “crouched” (exaggerating flexions) gait: wider support base, quicker double support phase and lower TBCM sway, in order to increase balance by improving ground clearance, reported in the literature (20). This gait profile is particularly used to compensate for a plantarflexed ankle (also known as foot drop), due to inadequate dorsiflexion control, which is a characteristic of the more advanced cases of nerve-length dependent neuropathy patients (29).

The obtained results are interestingly consistent with descriptions of the continuous disturbance of parkinsonian gait: slow, small-stepped, flexed walking pattern (30). Patients with diabetic neuropathy also walk in a more conservative way (31), which is in line with our findings. They present a reduction in medial–lateral and anterior–posterior displacement of the TBCM during walking (32), which is consistent with the loss of proprioception sensory feedbacks from the lower extremities and weakness of ankle plantar flexor and dorsiflexor muscles (31). Less dorsiflexion and increased plantar flexion were associated with a decrease in muscle strength of the foot flexors, both dorsi- and plantar-flexors, which may affect gait speed (33).

A study performed with diabetic patients, over 65 years old, walking at three different speeds showed that alterations in the gait of neuropathic patients were mainly related to reduced walking speed (34). They argue that the peripheral sensory loss affects gait variability, causing stride-to-stride alterations in the mediolateral and anteroposterior planes that reflect in stride width and stride time/length fluctuations, respectively (20, 34). This gait abnormality is likely to be present when there is a deficiency in the sensation or proprioception of the legs (20). Courtemanche et al. argued that the reduction in walking speed was, *per se*, a compensatory strategy to improve gait stability (35). Although we have analyzed the difference in gait speed variability between HC and SP groups, and it was not statistically significant, the reduced stride (or gait) speed and increased base (or step) width are supported by the present work.

When comparing the AC and SPS groups, the maximum elbow and minimum knee angles are the only with no statistically significant difference. From the parameters that present a significant difference, the temporal parameters have a higher mean value for the SPS group, while the value for spatio-temporal and kinematic parameters is overall lower. It is understandable once the SPS group is composed of patients at the onset or at the beginning of the disease development, with clinical involvement of small fibers and neuropathic pain in the feet and legs for some of them.

It is interesting to note that the trend (increase or decrease) of the mean value for most gait parameters is inverted when comparing the AC-SPS pair to the SPS-SPSL pair (when the mean value is higher for SPS than AC, it is lower for SPSL than SPS, and vice versa). This finding may be explained by the reported effect of protection of disease progression on the first years of early-stage treatment with tafamidis (most of the patients in the SPS group take this medication), in contrast to its use at a later time as well as in later-stage patients when it is less effective (36–38).

Looking at Table 2, it seems that the spatiotemporal parameters differences are more evident when comparing patients with neuropathic pain (especially SPS and SPSL). This may also be due to the anticipation or fear of pain, which have been associated with walking deficits (24), where the stride is lengthier. This has been called “antalgic gait,” which is assumed in order to avoid or lessen pain (39). The used of a pain clinical scale (or other measurement tool) would be useful in the evaluation of these groups.

It is possible to perceive from Table 3 that there are 15 parameters that may be used to distinguish between groups for all the group pairs (HC-AC, AC-SP, AC-SPS, SPS-SPSL, and SPSL-SMP). The differences in hip and ankle angle ranges have roughly 10 degrees of variation, or less, which are not high enough to be noticed by visual inspection, demonstrating the value that quantitative analysis of gait using a single RGB-D camera can have.

Although the statistically significant differences found are not directly correlated to any clinical feature already described, these are promising results in terms of future contributions to the onset detection and/or disease progression evaluation, since we can already perceive that there are recognizable differences between subjects without the V30M mutation and with the mutation (either with or without symptoms). It is important, nonetheless, to consider the limitations of this study, including the small sample size for the asymptomatic carriers and the patient subgroups, which reduces the statistical meaning of the analysis involving those groups, as well as the possible patients' stress associated to data collection, which may have contributed to additional gait alterations. Moreover, although the gait analysis protocol is not difficult or extensive, it is still not integrated in the normal activities of the hospital center, fact that can contribute to hinder data collection. Other limitation is the lack of neurophysiological examination (NCS, SSR, etc.) of the controls which could bring insight into the HC-AC analysis. Additionally, it would be interesting to study late-onset patients as well as other neuropathies to evaluate the diagnostic utility of the presented results. Despite these limitations, our results are promising and provide encouraging insight on using a RGB-D camera-based system to bring more objectivity to gait assessment in ATTRv V30M mutation carriers and support onset detection and patient follow-up. Nevertheless, more data is necessary to better understand the loss of gait ability and the degree of gait disturbance in these patients.

## Conclusions

Our main aim was to find if gait-related parameters obtained quantitatively could be used to distinguish between healthy subjects and ATTRv V30M mutation carriers (symptomatic and asymptomatic). We have concluded that several parameters can potentially be used to distinguish ATTRv V30M asymptomatic carriers from healthy controls, which indicates that asymptomatic carriers may have a subclinical gait change unnoticed to the naked eye that can be detected by quantitative gait analysis using an RGB-D camera system. If confirmed, this can contribute to an early access to treatment options and a consequent improvement of the patients' quality of life. We also found several parameters that can possibly be used to distinguish between different sub-groups of patients. A valuable application of this quantitative assessment is the longitudinal assessment of the patients, which may provide insights on inter-individual changes and help defining parameters that identify when a patient has clinically relevant neuropathy.

To the best of our knowledge, this is the first analysis of quantitative gait characteristics of ATTRv V30M mutation carriers. Furthermore, the used system has the major advantage of being non-intrusive, affordable and portable, being suitable for use in different clinical settings, without causing constraints to the patients nor difficulties to the clinical routine. This system could also be used to assess other neuropathies.

## Data Availability Statement

All datasets generated for this study are included in the article/supplementary material.

## Ethics Statement

The studies involving human participants were reviewed and approved by Ethics Committee of the Centro Hospitalar Universitário do Porto. The patients/participants provided their written informed consent to participate in this study.

## Author Contributions

The study was designed by MV-B, TC, and JC. MV-B and AR performed data collection and data processing. MV-B wrote the original draft. MC supervised patient selection. JC, TC, and JF supervised all work. All authors edited the manuscript, and read and approved the final manuscript.

## Conflict of Interest

TC and MC received support from Pfizer, which manufactures Tafamidis, to attend to scientific meetings and to integrate the speakers' bureau of Pfizer and received honoraria. TC and MC also received this support from IONIS Pharmaceuticals and Alnylam pharmaceuticals. The remaining authors declare that the research was conducted in the absence of any commercial or financial relationships that could be construed as a potential conflict of interest.

## References

[1] Schmidt HH Waddington-Cruz M Botteman MF Carter JA Chopra AS Hopps M. Estimating the global prevalence of transthyretin familial amyloid polyneuropathy. Muscle Nerve. (2018) 57:829–37. 10.1002/mus.26034 29211930 PMC5947118

[2] Ines M Coelho T Conceicao I Duarte-Ramos F de Carvalho M Costa J. Epidemiology of transthyretin familial amyloid polyneuropathy in portugal: a nationwide study. Neuroepidemiology. (2018) 51:177–82. 10.1159/000490553 30153683

[3] Parman Y Adams D Obici L Galan L Guergueltcheva V Suhr OB. Sixty years of transthyretin familial amyloid polyneuropathy (TTR-FAP) in Europe: where are we now? A European network approach to defining the epidemiology and management patterns for TTR-FAP. Curr Opin Neurol. (2016) 29(Suppl 1):S3–13. 10.1097/WCO.0000000000000288 26734951 PMC4739317

[4] Coutinho P da Silva AL Lopes J Resende P.-E Silva AMD Resende LAL. Forty Years of Experience With Type 1 Amyloid Neuropathy: Review of 483 Cases. Amsterdam: Excerpta Medica (1980).

[5] Mariani LL Lozeron P Theaudin M Mincheva Z Signate A Ducot B. Genotype-phenotype correlation and course of transthyretin familial amyloid polyneuropathies in France. Ann Neurol. (2015) 78:901–16. 10.1002/ana.24519 26369527 PMC4738459

[6] Adams D Koike H Slama M Coelho T. Hereditary transthyretin amyloidosis: a model of medical progress for a fatal disease. Nat Rev Neurol. (2019) 15:387–404. 10.1038/s41582-019-0210-4 31209302

[7] Ando Y Coelho T Berk JL Cruz MW Ericzon B.-G Ikeda S.-i. Guideline of transthyretin-related hereditary amyloidosis for clinicians. Orphanet J Rare Dis. (2013) 8:31. 10.1186/1750-1172-8-31 23425518 PMC3584981

[8] Andrade C. A peculiar form of peripheral neuropathy—familial atypical generalized amyloidosis with special involvement of the peripheral nerves. Brain. (1952) 75:408–27. 10.1093/brain/75.3.408 12978172

[9] Escolano-Lozano F Barreiros AP Birklein F Geber C. Transthyretin familial amyloid polyneuropathy (TTR-FAP): parameters for early diagnosis. Brain Behav. (2018) 8:e00889. 10.1002/brb3.889 29568686 PMC5853640

[10] Plante-Bordeneuve V. Transthyretin familial amyloid polyneuropathy: an update. J Neurol. (2018) 265:976–83. 10.1007/s00415-017-8708-4 29249054

[11] Benson MD Waddington-Cruz M Berk JL Polydefkis M Dyck PJ Wang AK. Inotersen treatment for patients with hereditary transthyretin amyloidosis. N Engl J Med. (2018) 379:22–31. 10.1056/NEJMoa1716793 29972757 PMC12611561

[12] Adams D Gonzalez-Duarte A O'Riordan WD Yang CC Ueda M Kristen A. Patisiran, an RNAi therapeutic, for hereditary transthyretin amyloidosis. N Engl J Med. (2018) 379:11–21. 10.1056/NEJMoa1716153 29972753

[13] Han J Shao L Xu D Shotton J. Enhanced computer vision with Microsoft Kinect sensor: a review. IEEE Trans Cybern. (2013) 43:1318–34. 10.1109/TCYB.2013.2265378 23807480

[14] Clark RA Mentiplay BF Hough E Pua YH. Three-dimensional cameras and skeleton pose tracking for physical function assessment: A review of uses, validity, current developments and Kinect alternatives. Gait Posture. (2018) 68:193–200. 10.1016/j.gaitpost.2018.11.029 30500731

[15] Vilas-Boas MdC Rocha AP Choupina H Cardoso MN Fernandes JM Coelho T Cunha JPS. Validation of a single RGB-D camera for gait assessment of polyneuropathy patients. Sensors. (2019) 19:4929 10.3390/s19224929 31726742 PMC6891607

[16] Vilas-Boas MdC Rocha AP Choupina HMP Cardoso M Fernandes JM Coelho T. TTR FAP Progression Evaluation Based on Gait Analysis Using a Single RGB D Camera. In: *41st Annual International Conference of the IEEE Engineering in Medicine and Biology Society (EMBC)*. Berlin, Germany, IEEE (2019). 10.1109/EMBC.2019.8857354 31947098

[17] Stone EE Skubic M. Passive In-Home Measurement of Stride-to-Stride Gait Variability Comparing Vision and Kinect Sensing. In: *33rd Annual International Conference of the IEEE EMBS*. Boston, MA (2011). 10.1109/IEMBS.2011.6091602 22255825

[18] Galna B Barry G Jackson D Mhiripiri D Olivier P Rochester L. Accuracy of the Microsoft Kinect sensor for measuring movement in people with Parkinson's disease. Gait and Posture. (2014) 39:1062–8. 10.1016/j.gaitpost.2014.01.008 24560691

[19] Rocha AP Choupina HMP Vilas-Boas MC Fernandes JM Cunha JPS. System for automatic gait analysis based on a single RGB-D camera. PLoS ONE. (2018) 13:e0201728. 10.1371/journal.pone.0201728 30075023 PMC6075757

[20] Whittle MW. Gait Analysis An Introduction. Amsterdam: Elsevier Ltd. (2007).

[21] Winter DA. Biomechanics and Motor Control of Human Movement. Hoboken, NJ: Wiley (2009).

[22] Wallis WHK. Use of ranks in one-criterion variance analysis. J Am Stat Assoc. (1952) 47:583–621. 10.1080/01621459.1952.10483441

[23] Conover WJ. Practical Nonparametric Statistics. 3rd edition. Hoboken, NJ: Wiley (1999).

[24] Al-Obaidi SM Al-Zoabi B Al-Shuwaie N Al-Zaabie N Nelson RM. The influence of pain and pain-related fear and disability beliefs on walking velocity in chronic low back pain. Int J Rehabil Res. (2003) 26:101–8. 10.1097/01.mrr.0000070750.63544.06 12799603

[25] Michalak J Troje NF Fisher J Vollmar P Heidenreich T Schulte D. embodiment of sadness and depression—gait patterns associated with dysphoric mood. Psychosom Med. (2009) 71:580–7. 10.1097/PSY.0b013e3181a2515c 19414617

[26] Gross MM Crane EA Fredrickson BL. Effort-shape and kinematic assessment of bodily expression of emotion during gait. Hum Mov Sci. (2012) 31:202–21. 10.1016/j.humov.2011.05.001 21835480

[27] Lopes A Fonseca I Sousa A Rodrigues C Branco M Coelho T. Psychopathological dimensions in subjects with hereditary ATTR V30M amyloidosis and their relation with life events due to the disease. Amyloid. (2018) 25:26–36. 10.1080/13506129.2018.1428795 29357699

[28] Ledo S Leite A Souto T Dinis MA Sequeiros J. Mid- and long-term anxiety levels associated with presymptomatic testing of Huntington's disease, Machado-Joseph disease, and familial amyloid polyneuropathy. Braz J Psychiatry. (2016) 38:113–20. 10.1590/1516-4446-2014-1617 26870910 PMC7111364

[29] Sekijima Y. Hereditary transthyretin amyloidosis. In: Adam MP, Ardinger HH, Pagon RA, Wallace SE, Bean LJH, Stephens K, editors, et al. GeneReviews®. Seattle, WA: University of Washington 1993–2019 (2001).

[30] Hausdorff JM. Gait dynamics in Parkinson's disease: common and distinct behavior among stride length, gait variability, and fractal-like scaling. Chaos. (2016) 19:026113. 10.1063/1.3147408 19566273 PMC2719464

[31] Mustapa A Justine M Mohd Mustafah N Jamil N Manaf H. Postural control and gait performance in the diabetic peripheral neuropathy: a systematic review. Biomed Res Int. (2016) 2016:9305025. 10.1155/2016/9305025 27525281 PMC4971307

[32] Grewa GS Bharara M Menzies R Talal TK Armstrong D Najafi B. Diabetic peripheral neuropathy and gait: does footwear modify this association? J Diabetes Sci Technol. (2013) 7:1138–46. 10.1177/193229681300700506 24124939 PMC3876356

[33] Martinelli AR Mantovani AM Nozabieli AJ Ferreira DM Barela JA Camargo MR. Muscle strength and ankle mobility for the gait parameters in diabetic neuropathies. Foot. (2013) 23:17–21. 10.1016/j.foot.2012.11.001 23274122

[34] Wuehr M Schniepp R Schlick C Huth S Pradhan C Dieterich M. Sensory loss and walking speed related factors for gait alterations in patients with peripheral neuropathy. Gait Posture. (2014) 39:852–8. 10.1016/j.gaitpost.2013.11.013 24342450

[35] Courtemanche R Teasdale N Boucher P Fleury M Lajoie Y Bard C. Gait problems in diabetic neuropathic patients. Arch Phys Med Rehabil. (1996) 77:849–55. 10.1016/S0003-9993(96)90269-5 8822673

[36] Coelho T Maia LF da Silva AM Cruz MW Plante-Bordeneuve V Suhr OB. Long-term effects of tafamidis for the treatment of transthyretin familial amyloid polyneuropathy. J Neurol. (2013) 260:2802–14. 10.1007/s00415-013-7051-7 23974642 PMC3825212

[37] Cortese A Vita G Luigetti M Russo M Bisogni G Sabatelli M. Monitoring effectiveness and safety of Tafamidis in transthyretin amyloidosis in Italy: a longitudinal multicenter study in a non-endemic area. J Neurol. (2016) 263:916–24. 10.1007/s00415-016-8064-9 26984605

[38] Monteiro C Mesgazardeh JS Anselmo J Fernandes J Novais M Rodrigues C. Predictive model of response to tafamidis in hereditary ATTR polyneuropathy. JCI Insight. (2019) 4:e126526. 10.1172/jci.insight.126526 31217346 PMC6629131

[39] Lalli P Chan A Garven A Midha N Chan C Brady S. Increased gait variability in diabetes mellitus patients with neuropathic pain. J Diabetes Complications. (2013) 27:248–54. 10.1016/j.jdiacomp.2012.10.013 23218484

