# Corrigendum: Clinical 3-D gait assessment of patients with polyneuropathy associated with hereditary transthyretin amyloidosis

**DOI:** 10.3389/fneur.2022.980597

**Published:** 2022-08-17

**Authors:** Maria do Carmo Vilas-Boas, Ana Patrícia Rocha, Márcio Neves Cardoso, José Maria Fernandes, Teresa Coelho, João Paulo Silva Cunha

**Affiliations:** ^1^INESC TEC, FEUP and LABIOMEP, University of Porto, Porto, Portugal; ^2^Unidade Corino de Andrade and Neurophysiology Department, Centro Hospitalar Universitário Do Porto, Porto, Portugal; ^3^Institute of Electronics and Informatics Engineering of Aveiro (IEETA), Department of Electronics, Telecommunications and Informatics, University of Aveiro, Aveiro, Portugal

**Keywords:** ATTRv V30M, amyloidosis, polyneuropathy, gait, quantitative assessment, ambulatory, markerless vision-based systems

In the published article, there was an error in Table 2 as published. The units of the Total body center of mass sway in *x-axis* (TBCMx) and y-axis (TBCMy) were shown in mm when they should be in cm. The corrected Table 2 and its caption appear below.

**Table 2:** Mean ± standard deviation values for each gait parameter and each subject group (1. Healthy Controls – HC; 2. Asymptomatic Carriers – AC; 3. Symptomatic Patients – SP; 3.1 Patients with small-fiber sensory polyneuropathy – SPS – and 3.2 with large-fiber sensory polyneuropathy – SPSL; and 3.3 Patients with motor neuropathy – SMP).

**Gait parameter**	**1. HC**	**2. AC**	**3. SP**	**3.1 SPS**	**3.2 SPSL**	**3.3 SMP**
Stride duration, s	1.238 ± 0.386	1.463 ± 0.518	1.628 ± 0.690	1.709 ± 0.729	1.463 ± 0.509	1.616 ± 0.768
Stride length, cm	114.7 ± 23.0	101.5 ± 28.0	93.6 ± 25.5	92.8 ± 25.6	91.7 ± 23.7	100.2 ± 27.3
Step duration, s	0.626 ± 0.282	0.732 ± 0.343	0.823 ± 0.489	0.862 ± 0.517	0.744 ± 0.396	0.816 ± 0.513
Step length, cm	52.2 ± 13.5	47.3 ± 15.5	42.6 ± 15.6	42.6 ± 15.9	41.6 ± 15.3	44.7 ± 14.4
Step width, cm	12.4 ± 3.7	12.1 ± 3.8	13.0 ± 4.2	13.7 ± 4.4	12.1 ± 4.1	12.2 ± 3.2
Stance duration, s	0.784 ± 0.261	0.943 ± 0.396	1.012 ± 0.525	1.057 ± 0.559	0.901 ± 0.360	1.044 ± 0.608
Swing duration, s	0.455 ± 0.245	0.521 ± 0.301	0.616 ± 0.414	0.652 ± 0.451	0.562 ± 0.342	0.573 ± 0.367
Single support duration, s	0.897 ± 0.331	1.043 ± 0.451	1.219 ± 0.575	1.292 ± 0.623	1.104 ± 0.440	1.142 ± 0.559
Double support duration, s	0.341 ± 0.125	0.421 ± 0.172	0.409 ± 0.267	0.417 ± 0.296	0.359 ± 0.139	0.474 ± 0.309
Gait speed, m/s	1.047 ± 0.239	0.846 ± 0.228	0.728 ± 0.180	0.696 ± 0.161	0.767 ± 0.182	0.785 ± 0.218
Gait speed variability, m/s	0.105 ± 0.057	0.153 ± 0.384	0.121 ± 0.216	0.107 ± 0.119	0.125 ± 0.208	0.170 ± 0.423
Foot swing velocity, m/s	2.679 ± 1.031	2.318 ± 1.334	1.894 ± 1.109	1.807 ± 1.103	1.917 ± 0.917	2.195 ± 1.376
Arm swing velocity, m/s	1.976 ± 0.749	1.570 ± 0.798	1.384 ± 0.709	1.312 ± 0.515	1.405 ± 0.575	1.633 ± 1.299
Total body center of mass sway in *x-axis*, cm	29.2 ± 38.7	35.5 ± 26.1	34.1 ± 37.9	29.7 ± 33.1	44.7 ± 41.5	31.5 ± 44.2
Total body center of mass sway in *y-axis*, cm	10.2 ± 5.4	13.7 ± 15.5	11.1 ± 8.4	10.9 ± 7.5	11.6 ± 10.7	10.9 ± 7.0
Neck angle, deg	166.1 ± 10.2	162.3 ± 14.2	158.1 ± 17.7	160.4 ± 13.8	152.1 ± 23.5	160.2 ± 16.5
Spine shoulder angle, deg	171.3 ± 5.8	168.5 ± 8.7	165.7 ± 11.3	167.1 ± 8.9	161.9 ± 15.3	167.2 ± 9.4
Spine middle angle, deg	175.9 ± 2.2	174.7 ± 2.7	173.6 ± 3.3	173.8 ± 3.2	172.8 ± 3.6	173.9 ± 3.3
Maximum elbow angle, deg	167.6 ± 10.9	166.0 ± 9.7	166.0 ± 10.1	166.1 ± 9.3	166.5 ± 12.8	164.5 ± 6.2
Minimum elbow angle, deg	144.3 ± 20.4	144.7 ± 20.4	144.8 ± 21.8	145.3 ± 20.1	144.7 ± 26.0	142.6 ± 19.9
Maximum knee angle, deg	174.5 ± 3.1	176.8 ± 2.5	174.8 ± 3.5	174.5 ± 3.6	174.8 ± 3.4	176.1 ± 3.1
Minimum knee angle, deg	142.0 ± 17.7	142.5 ± 17.1	143.1 ± 18.4	142.6 ± 18.1	145.3 ± 17.7	141.1 ± 20.8
Hip angle range, deg	19.3 ± 7.0	18.9 ± 7.5	17.1 ± 6.8	17.3 ± 7.2	16.0 ± 5.8	18.4 ± 7.1
Ankle angle range, deg	33.6 ± 16.6	27.9 ± 17.6	20.7 ± 16.0	17.6 ± 14.2	22.0 ± 16.0	30.3 ± 18.4

In the published article, there was an error in Table 3 as published. The units of the Total body center of mass sway in *x-axis* (TBCMx) and y-axis (TBCMy) were shown in mm. The correct unit is cm. The corrected Table 3 and its caption appear below.

**Table 3:** Results of the Conover-Iman test (*p*-value) pairwise comparison between the six groups included in Table 2 (HC, AC, SP, SPS, SPSL and SMP), for each gait parameter.

**Gait parameter**	**HC–AC**	**HC–SP**	**AC–SP**	**AC–SPS**	**SPS–SPSL**	**SPSL–SMP**	**HC–SMP**
Stride duration, s	≤ 0.001	≤ 0.001	≤ 0.001	≤ 0.001	≤ 0.001	≤ 0.001	≤ 0.001
Stride length, cm					0.04		
Step duration, s					≤ 0.001		
Step length, cm							
Step width, cm	N.S.					N.S.	N.S.
Stance duration, s	≤ 0.001					≤ 0.001	≤ 0.001
Swing duration, s						N.S.	
Single support duration, s							
Double support duration, s						≤ 0.001	
Gait speed, m/s						N.S.	
Gait speed variability, m/s		N.S.				≤ 0.001	
Foot swing velocity, m/s		≤ 0.001				0.002	
Arm swing velocity, m/s						≤ 0.001	
Total body center of mass sway in *x-axis* (TBCMx), cm							0.036
Total body center of mass sway in *y-axis* (TBCMy), cm	N.S.				N.S.		N.S.
Neck angle, deg	≤ 0.001				≤ 0.001		≤ 0.001
Spine shoulder angle, deg							
Spine middle angle, deg							
Maximum elbow angle, deg			N.S.	N.S.			
Minimum elbow angle, deg	N.S.		0.009	0.038	0.003		N.S.
Maximum knee angle, deg	≤ 0.001		≤ 0.001	≤ 0.001	0.046		≤ 0.001
Minimum knee angle, deg	0.007		N.S.	N.S.	0.002	0.022	N.S.
Hip angle range, deg	≤ 0.001		≤ 0.001	≤ 0.001	≤ 0.001	≤ 0.001	≤ 0.001
Ankle angle range, deg							

N.S. stands for non-significant (p-value > 0.05).

In the published article, there was an error in Figure 3 as published. The units of the Total body center of mass sway in x-axis were shown in mm in the vertical axis of the plot. The correct unit is cm. The corrected Figure 3 and its caption appear below.

**Figure 3:**
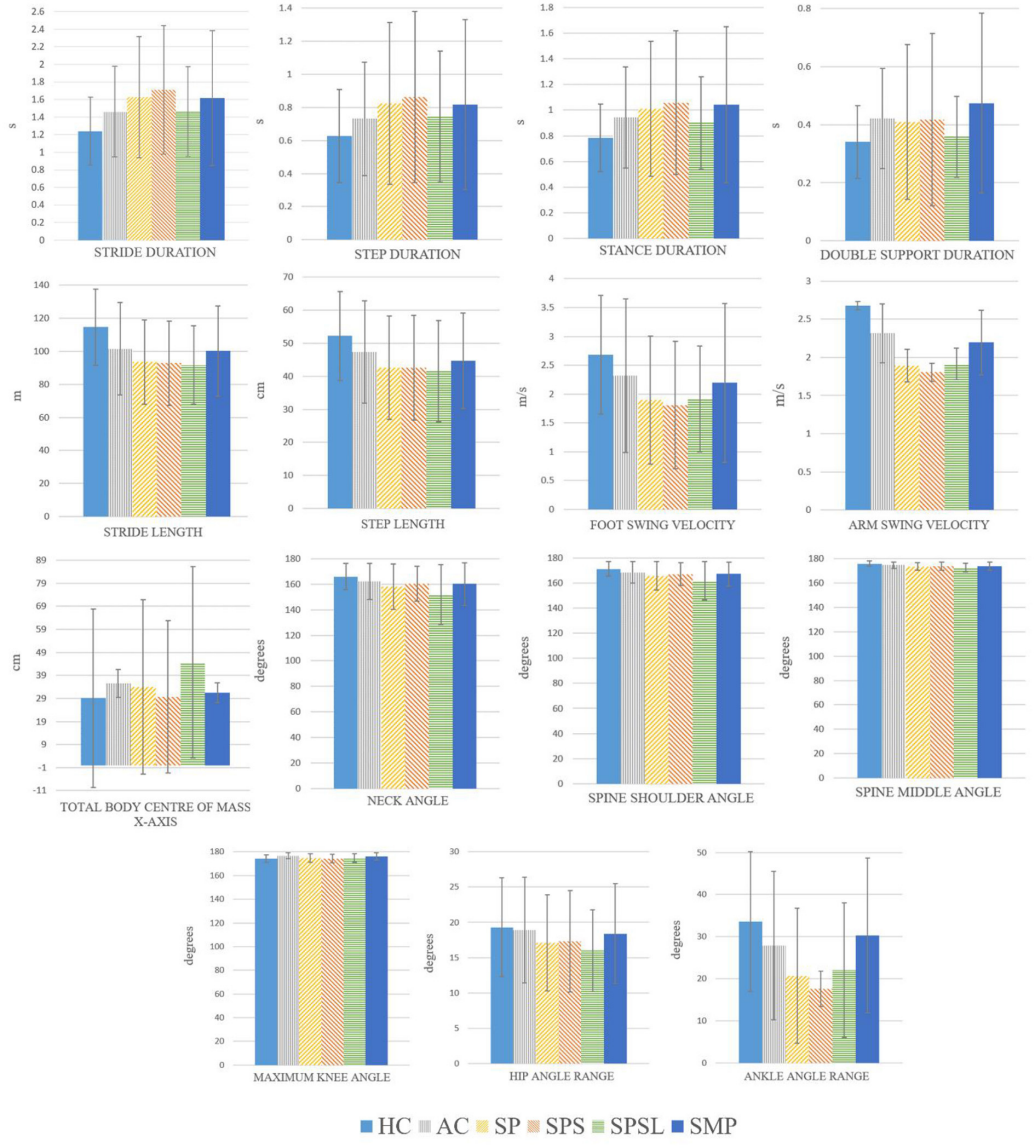
Mean and standard deviation for each subject group, for the gait parameters that showed statistically significant differences in the Conover-Iman test (p ≤ 0.05) for all the comparisons, when comparing different pairwise comparison, included in Table III, between the six groups (HC, AC, SP, SPS, SPSL and SMP).

In the published article, there was an error in Supplementary Table S.I. The units of the Total body center of mass sway in *x-axis* (TBCMx) and y-axis (TBCMy) were shown in mm. The correct unit is cm. The correct material statement appears below.

**Table S.I:** Results of the Conover-Iman test (*p*-value) for the pairwise comparisons not presented in Table 3 between the six considered groups (HC, AC, SP, SPS, SPSL and SMP), for each gait parameter.

**Gait parameter**	**HC-SPS**	**HC–SPSL**	**AC–SPSL**	**AC–SMP**	**SPS–SMP**
Stride duration, s	≤ 0.001	≤ 0.001	N.S.	≤ 0.001	≤ 0.001
Stride length, cm			≤ 0.001	0.003	
Step duration, s			N.S.	≤ 0.001	0.005
Step length, cm			≤ 0.001		N.S.
Step width, cm		N.S.	0.035	N.S.	≤ 0.001
Stance duration, s		≤ 0.001	≤ 0.001	≤ 0.001	N.S.
Swing duration, s					≤ 0.001
Single support duration, s					
Double support duration, s				N.S.	
Gait speed, m/s				≤ 0.001	
Gait speed variability, m/s	N.S.	0.009		N.S.	
Foot swing velocity, m/s	≤ 0.001	≤ 0.001		0.021	
Arm swing velocity, m/s				N.S.	
Total body center of mass sway in *x-axis* (TBCMx), cm			0.015	≤ 0.001	
Total body center of mass sway in *y-axis* (TBCMy), cm			≤ 0.001	N.S.	0.023
Neck angle, deg				N.S.	≤ 0.001
Spine shoulder angle, deg				0.03	N.S.
Spine middle angle, deg				0.003	
Maximum elbow angle, deg		N.S.		≤ 0.001	
Minimum elbow angle, deg		≤ 0.001		0.021	≤ 0.001
Maximum knee angle, deg	0.037			≤ 0.001	
Minimum knee angle, deg	0.004			N.S.	
Hip angle range, deg	≤ 0.001				N.S.
Ankle angle range, deg				0.025	0.01

N.S. stands for non-significant (p-value > 0.05).

In the published article, there was a mistake on the computation description of one of the assessed parameters (total body center of mass). A correction has been made to “*Data Processing*,” Paragraph 3:

“For each gait cycle, we computed the 24 spatiotemporal and kinematic gait parameters listed in Table 2 and defined in (15). The total body center of mass (TBCM) sway was computed as the standard deviation of the distance (in the x/y-axis, i.e., medial-lateral and vertical directions) of the total body center of mass (TBCM), in relation to the RGB-D sensor's coordinate system, for all gait cycle frames. For each frame, TBCM's position is the mean position of all body segments' CM, which was obtained according to (21).”

The authors apologize for these errors and state that this does not change the scientific conclusions of the article in any way. The original article has been updated.

## Publisher's note

All claims expressed in this article are solely those of the authors and do not necessarily represent those of their affiliated organizations, or those of the publisher, the editors and the reviewers. Any product that may be evaluated in this article, or claim that may be made by its manufacturer, is not guaranteed or endorsed by the publisher.

