# Supplementary material for: Clinical 3-D Gait Assessment of Patients With Polyneuropathy Associated With Hereditary Transthyretin Amyloidosis
Source: Front Neurol. 2020 Nov 23;11:605282. doi: 10.3389/fneur.2020.605282 (PMC7719818; doi:10.3389/fneur.2020.605282)
Supplement: Supplementary file 1 [file Table_1.docx]

Supplementary Material

**Table S.I.** Results of the Conover-Iman test (*p*-value) for the pairwise comparisons not presented in Table 3 between the six considered groups (HC, AC, SP, SPS, SPSL and SMP), for each gait parameter.

| **Gait Parameter** | **HC-SPS** | **HC–SPSL** | **AC–SPSL** | **AC–SMP** | **SPS–SMP** |
| --- | --- | --- | --- | --- | --- |
| **Stride duration, s** | ≤ 0.001 | ≤ 0.001 | N.S. | ≤ 0.001 | ≤ 0.001 |
| **Stride length, cm** |  |  | ≤ 0.001 | 0.003 |  |
| **Step duration, s** |  |  | N.S. | ≤ 0.001 | 0.005 |
| **Step length, cm** |  |  | ≤ 0.001 |  | N.S. |
| **Step width, cm** |  | N.S. | 0.035 | N.S. | ≤ 0.001 |
| **Stance duration, s** |  | ≤ 0.001 | ≤ 0.001 | ≤ 0.001 | N.S. |
| **Swing duration, s** |  |  |  |  | ≤ 0.001 |
| **Single support duration, s** |  |  |  |  |  |
| **Double support duration, s** |  |  |  | N.S. |  |
| **Gait speed, m/s** |  |  |  | ≤ 0.001 |  |
| **Gait speed variability, m/s** | N.S. | 0.009 |  | N.S. |  |
| **Foot swing velocity, m/s** | ≤ 0.001 | ≤ 0.001 |  | 0.021 |  |
| **Arm swing velocity, m/s** |  |  |  | N.S. |  |
| **Total body center of mass sway in *x-axis* (TBCMx), cm** |  |  | 0.015 | ≤ 0.001 |  |
| **Total body center of mass sway in *y-axis* (TBCMy), cm** |  |  | ≤ 0.001 | N.S. | 0.023 |
| **Neck angle, deg** |  |  |  | N.S. | ≤ 0.001 |
| **Spine shoulder angle, deg** |  |  |  | 0.03 | N.S. |
| **Spine middle angle, deg** |  |  |  | 0.003 |  |
| **Maximum elbow angle, deg** |  | N.S. |  | ≤ 0.001 |  |
| **Minimum elbow angle, deg** |  | ≤ 0.001 |  | 0.021 | ≤ 0.001 |
| **Maximum knee angle, deg** | 0.037 |  |  | ≤ 0.001 |  |
| **Minimum knee angle, deg** | 0.004 |  |  | N.S. |  |
| **Hip angle range, deg** | ≤ 0.001 |  |  |  | N.S. |
| **Ankle angle range, deg** |  |  |  | 0.025 | 0.01 |

N.S. stands for non-significant (*p*-value > 0.05).
